# Dissection of Agonistic and Blocking Effects of CD200 Receptor Antibodies

**DOI:** 10.1371/journal.pone.0063325

**Published:** 2013-05-14

**Authors:** Munir Akkaya, Marie-Laure Aknin, Billur Akkaya, A. Neil Barclay

**Affiliations:** 1 Sir William Dunn School of Pathology, University of Oxford, South Parks Road, Oxford, United Kingdom; 2 Nuffield Department of Clinical Medicine, University of Oxford, Oxford, United Kingdom; St. Jude Children’s Research Hospital, United States of America

## Abstract

The CD200 receptor (CD200R) is present mainly on myeloid cells and gives inhibitory signals when engaged by its ligand CD200. The interaction is currently of therapeutic interest in cancer and inflammation. However functional effects are complicated by the fact that CD200R is itself polymorphic and also a member of a paired receptor family with four closely related gene products in mice called CD200RLa etc. We show that a second allele of CD200R (termed CD200R(2)) that differs in 7 amino acids also binds CD200 but did not react with the widely used CD200R antibody OX110. Biochemical and functional analysis showed that the CD200/CD200R interaction was blocked by the OX131, mAb that recognises both CD200R(1) and CD200R(2), but not by OX110 mAb. Both mAb can give agonistic inhibitory signals but functional analysis shows OX131 mAb also has the potential to block inhibition by preventing the ligand-receptor interaction and hence gives opposing effects. Although OX131 mAb cross-reacts with the activating receptor CD200RLe, it is specific for CD200R in C57BL/6 whilst OX110 mAb cross-reacts on CD200RLc. The results show the importance of the repertoire of paired receptors in strains or individuals and mAb used with implications for paired receptor analysis and therapeutics.

## Introduction

CD200R is a member of a paired receptor family consisting of membrane proteins that have closely related extracellular regions but differing cytoplasmic regions so that members may give opposite types of signals [1]. The activating members have short cytoplasmic domains and associate with adaptors such as DAP12 which contain immunoreceptor tyrosine-based activation motifs (ITAM) [1], [2], [3]. Most inhibitory receptors contain immunoreceptor tyrosine based inhibitory motifs (ITIM) that recruit phosphatases but CD200R is unusual in containing a phosphotyrosine motif that recruits the adaptors DOK2 and RasGAP leading to inhibition of the ERK pathway [3], [4], [5]. CD200R is expressed on a variety of leukocytes and in particular myeloid cells such as macrophages [1]. CD200R binds a broadly distributed membrane protein CD200 [1], [6]. In this respect it has similarities to the SIRP paired receptor family where SIRPα also binds a widely distributed membrane protein CD47 [7]. Another similarity is that both SIRPα and CD200R interactions are the subject of interest as possible therapeutics especially for cancer [8], [9], [10], [11], [12]. One complication is that paired receptors are frequently characterised by varying numbers of members and by a high degree of polymorphism [13] that may lead to unexpected results according to the fine specificity of the reagents.

**Figure 1:**
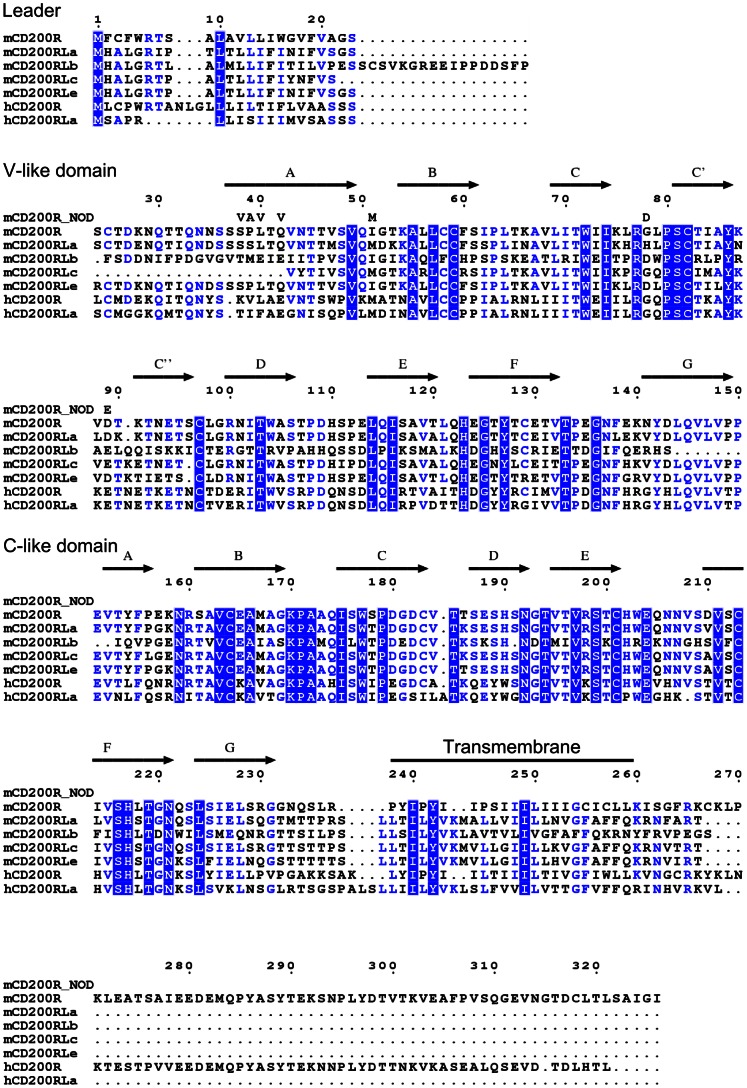
Sequence alignment of CD200R and CD200R like proteins. Residues identical in all sequences are highlighted in blue, residues identical in 5 or 6 sequences are in blue font. The superscript bars predict the extent of the beta strands characteristic of the Ig fold by comparison with solved structures. The accession numbers from UNIPROT for mouse sequences are mCD200R (also termed CD200R(1)) from C57BL/6 mice (Q9ES57), mCD200RLa (Q6XJV4**)**, mCD200RLb (Q5UKY4), mCD200RLc (Q6XJV6), mCD200RLe (Q8BTP3) and for human sequences hCD200R (Q8TD46) and hCD200RLa (Q6Q8B3). 7 amino acid differences have been identified in domain one in the NOD mice compared to the that in C57BL/6 mice and these are indicated by mCD200R_NOD (also termed CD200R(2)) [16].

In humans there is one potential activating member, CD200RLa but in mice there are four activating members, CD200RLa, CD200RLb, CD200RLc and CD200RLe. (An alternative nomenclature is also used, CD200R4, CD200R3, CD200R2 and CD200R5 respectively [14], [15]). The extracellular domains of the activating members CD200R family share up to 87% amino acid sequence identity with the inhibitory receptor but do not bind CD200 (Fig. 1) [15]. These genes are not present in all mice strains; C57BL/6, BALB/c and B10 that possess CD200RLc do not have CD200RLe which in turn is present on NOD, AKR and CD1 mice [16]. The activating CD200R members show a more restricted distribution than CD200R [1], [2]. Two alleles of CD200R differing by seven amino acids in their extracellular region are present in similar numbers of strains [16]. With so much variability in both gene number and sequence it is difficult to get specific reagents; hence it is likely that different results may be obtained with the same reagents in different mice strains. This may be a common phenomenon for paired receptors with similar levels of heterogeneity in gene number and polymorphisms being found in the SIRP family where many reagents cross-react or recognize a subpopulation of alleles [17], [18].

We describe how the commonly used mAb OX110 recognises CD200R in only some mouse strains and cross-reacts on CD200RLc, and a new mAb (OX131) that sees CD200R from both alleles. Both CD200R mAb give agonistic signals but OX131 mAb had additional effects as it blocks ligand engagement. OX131 mAb does not cross-react on activating receptors in C57BL/6 mice enabling a definitive tissue distribution to be determined. We describe a new mAb (OX132) that recognises CD200RLc and analyse tissues for its expression.

## Materials and Methods

### Ethics

All procedures were carried out under the terms of the UK Animals (Scientific Procedures) Act Home Office Project Licence and were approved by the University of Oxford Animal Care and Ethical Review Committee. The mice were kept under specific pathogen free conditions.

### Cloning of CD200R Family and DAP12 Genes

Full length coding sequences (with accession numbers) of CD200R(1) (BC052682), CD200R(2) (BAE32516), CD200RLa (NM207244), CD200RLb (NM029018) and CD200RLe (BAC40774) were sub-cloned into the pFB-Neo retroviral vector (Stratagene) from constructs previously described in [1], [15], [16]. The full length coding sequence of CD200RLc was amplified using cDNA generated from C57BL/6 mouse peritoneal exudate cells and cloned into pFB-Neo. The coding region of mouse DAP12 (NM011662) distal to its leader sequence [19] was amplified from mouse peritoneal exudate cell cDNA and cloned into pEF-BOS vector [20] which had a previously inserted rCD4 leader and N-terminus FLAG™ tag sequence. The construct consisted of a rCD4 leader, FLAG™ tag and mouse DAP12 was sub-cloned into pFB-Hygro [21]. The full length coding sequence of CD200 (BC051984; Image Clone 6413363) was subcloned into pFB-Hygro. pEF-BOS constructs coding for soluble fusion proteins containing extracellular domains of members of the CD200R family attached to rat CD4 domains 3 and 4 (rCD4d3+4) and a biotinylation site are as [1], [15] except for CD200R(2) which was prepared with the extracellular domain of CD200R(2) equivalent to that of the CD200R(1) construct.

### Assay to Test for mAb Specificity

125 µg streptavidin coated magnetic beads (Dynabeads M-280 Streptavidin) (Invitrogen) were coated with 20 µl concentrated tissue culture supernatant containing the biotinylated recombinant protein and incubated with rotation at 4°C overnight. The beads were washed three times with PBS (1% BSA and 10 mM NaN_3_), stained with antibodies and assayed by flow cytometry.

### Primary Cells

Mouse T, B, and NK cell populations were isolated from the spleens of C57BL/6 and BALB/c mice. Splenocytes were also cultured *in vitro* and stimulated for 48–72 hours with 1000 U IL-2 or 1 µg/ml LPS or plate bound CD3 mAb to get activated NK cell, B cell and T cell populations respectively [22], [23]. Resident tissue macrophages were isolated from peritoneal exudates. Neutrophils and inflammatory macrophages were isolated from mice previously injected with biogel or zymozan as in [24], [25]. Mast cells, basophils, dendritic cells and eosinophils were generated by culturing C57BL/6 bone marrow cells in IMDM (Invitrogen) media containing 15% fetal calf serum, 50 µM 2-mercaptoethanol, 50 U/ml penicillin, 50 µM streptomycin (PAA), 25 µg/ml gentamycin (Gibco), 2 mM L-glutamine (PAA), 0.1 mM non-essential amino acids (Sigma), 1 mM sodium pyruvate (Sigma), 10 mM HEPES (PAA) and by stimulating with the following conditions; for mast cells and basophils, cells were stimulated with SCF (Sigma) (50 ng/ml) and IL-3 (Sigma) (10 ng/ml) for four to six weeks as described [26]; for dendritic cells, stimulation with 10% X63-GMCSF cell line supernatant according to [27] followed by addition of 1 µg/ml LPS on final day to achieve a mature phenotype; for eosinophils, cells were stimulated with SCF (100 ng/ml) and Flt3-ligand (100 ng/ml) for four days, with 10% X63-GMCSF supernatant, IL-3 (20 ng/ml) and IL-5 (Peprotech) (10 ng/ml) for four days and with IL-3 (20 ng/ml) and IL-5 (10 ng/ml) for three days [28]. Splenocytes, peritoneal exudate cells and bone marrow cells were also prepared from CD200R knock out mice [29].

### Transfection and Transduction of Cell Lines

Soluble chimeric proteins, containing rCD4d3+4 and a biotinylation site and the extracellular domains of the molecule of interest, were produced by transfecting 293T cells with the pEF-BOS constructs using polyethylenimine [30]. After incubation at 37°C for 5–6 hours, the media was replaced with XVIVO 20 (Lonza) serum free media and incubated for 4–6 days. The tissue culture supernatants were harvested, concentrated and biotinylated as described previously [15], [31].

CD200R, CD200RL’s and CD200 were expressed on 2B4 Reay, RBL.2H3 and CHO-IE^k^ cells respectively by transduction using retroviruses generated by transfection of Phoenix Eco packaging cell line [32] and selection with G418 (500 µg/ml) (Sigma) and/or hygromycin (Sigma) (300 µg/ml) one day after transduction. The RBL cells are available from the European Collection of Cell Cultures (Salisbury UK). The other cells were from Marion H. Brown and described in [33].

### Production of Monoclonal Antibodies

mAb were produced against mouse CD200R and CD200RLc by immunisation of DA rats with recombinant protein consisting of the extracellular regions of these proteins together with rCD4d3+4 and attachment to streptavidin coated beads (as used in the analysis of mAb). After fusion to the Y3 hybridoma by standard procedures, the supernatants were screened on cell lines expressing the various gene products (see above). The rat anti-mouse CD200R and CD200RLc hybridomas were named OX131 and OX132 respectively.

### Antibodies, Staining Reagents and Flow Cytometry

OX110 (rat anti-mouse CD200R), OX131 (rat anti-mouse CD200R), OX132 (rat anti-mouse CD200RLc), OX90 (rat anti-mouse CD200), OX11 (rat anti-rat kappa chain) were purified using HiTrap Protein G HP (GE Healthcare) affinity chromatography from spent tissue culture media from hybridoma cell lines grown in bio-reactors (Integra Biosciences) in Hybridoma SFM (Invitrogen) media supplemented with penicillin, streptomycin, L-glutamine, 2-ME, sodium pyruvate and non-essential amino acids. Pacific Blue anti-mouse I-Ab, Pacific Blue anti-mouse CD45R/B220, PE/cy7 anti-mouse Gr1, FITC anti-mouse CD4, FITC anti-mouse CD49b, PERCP anti-mouse CD11c, PE/Cy5 anti-mouse CD3ε, PE/Cy7 anti-mouse NK 1.1, FITC anti-mouse CD11b, PERCP/Cy 5.5 anti-mouse FcεRIα were from Biolegend. Alexa Fluor 647 rat anti-mouse CD200R, FITC anti-rat IgG were from AbD Serotec. Alexa Fluor 647 rat IgG2a isotype control was from Invitrogen, PE/Cy 7 anti-mouse CD8 from eBioscience, PE anti-mouse Siglec F from BD Pharmingen and polyclonal IgG from rat serum from Sigma. BD Cytofix/Cytoperm plus kit (BD Pharmingen) was used during preparation of cells for intracellular staining with fluorescent antibodies.

The Fab fragment of OX131 and F(ab)’_2_ fragments of OX131, OX132 and polyclonal rat IgG were generated using kits from Pierce. F(ab)’_2_ fragments were purified by gel filtration and conjugated with PE or APC using LYNX rapid antibody conjugation kits (AbD Serotec).

Streptavidin coated yellow fluorescent beads and magnetic beads were from Spherotech and Invitrogen respectively.

Dead cells were excluded using LIVE/DEAD fixable dead cell stain kits (Invitrogen) and cell singlets were gated as outlined in [34]. Flow cytometry analyses were carried out using Dako CyAn™, BD FACsort™, BD FACSCalibur™ and data were analysed using FlowJo and CellQuest software.

### Characterization of CD200R mAb by Surface Plasmon Resonance (SPR)

Tissue culture supernatants containing biotinylated CD200R(1)-rCD4d3+4, CD200R(2)-rCD4d3+4 and rCD4d3+4 were immobilized onto flow cells at approximately equal response units (approximately 700 RU) to which streptavidin had been coupled [15]. Purified and gel filtered mouse CD200-rCD4d3+4 protein, OX110 and OX131 mAb were passed over the immobilized proteins and changes in CD200 binding (0.75 µM) were monitored. All experiments were performed using BIAcore 3000 at 25°C.

### Rat Basophilic Leukemia Degranulation Assay

Stable rat basophilic leukemia (RBL.2H3) cell lines expressing activating members of mouse CD200R family together with mouse DAP12 were tested for degranulation upon stimulation with mAb against the activating receptors. 2×10^5^ RBL.2H3 cells/200 µl complete RPMI (10% fetal calf serum, 50 U/ml penicillin-10 µg/ml streptomycin, 2 mM L-glutamine, 50 µM 2-ME) per well were plated onto flat bottom 96 well plates. After an overnight incubation at 37°C with 5% CO_2_, plates were put on ice and media were replaced with 50 µl/well primary antibody at 10 µg/ml in complete RPMI media. This step was carried out on ice and very slowly to prevent spontaneous degranulation of RBL.2H3 cells. This mixture was incubated overnight at 37°C, 5% CO_2_. The plates were put on ice, supernatants collected and transferred into a separate 96 well round bottom plate and the remaining cells were lysed using 50 µl 1% Triton X-100 (in PBS) with shaking. Cell lysates were collected and transferred to a round bottom 96 well plate. To evaluate the level of RBL.2H3 cell degranulation, 20 µl from each well of collected RBL.2H3 supernatant or lysate was mixed with 20 µl of substrate buffer (34 mg p-nitrophenyl N-acetyl β-D glucosaminide. (Sigma) and 0.2% (v/v) Triton-X-100 in 20 ml 50 mM sodium citrate, pH 4.8) on ice and transferred to 37°C for 1 hour incubation. The reaction was terminated by adding 200 µl 33 mM glycine, 83 mM Na_2_CO_3_, 67 mM NaCl and absorbance measuered at 405 nm. The percentage of degranulation was calculated using the formula “*Degranulation % = 100 x (O.D._(Supernatants)_/O.D._(Supernatants)_ +O.D._(Lysate)_ )*”.

### T cell Stimulation Model to Test mAb against CD200R

OX110, OX131 and OX90 mAb were tested for their ability to interfere with the CD200-CD200R interaction. CHO-IE^k^ –CD200 and 2B4 Reay-CD200R(1) stable cell lines in DMEM (10^5^ cells/50 µl) were mixed per well in round bottom 96 well plates. The final mixture contained 10^5^ CHO, 10^5^ 2B4 cells, 1 mM MCC and 10 µg/ml mAb in a total volume of 200 µl/well was incubated at 37°C for 16–24 hours and supernatants collected. The effect of mAb (OX110 and OX131) mediated receptor (CD200R) oligomerization was measured by coating round bottom 96 well plates with 10 µg/ml CD200R or control (OX132) mAb together with 0.5 µg/ml CD3 mAb (MC11). 2×10^5^/well 2B4 Reay-CD200R(1) stable cell lines were added to the coated plates, the plates were incubated and supernatants were collected as described above. IL-2 was assayed by ELISA (BD Biosciences).

### Statistical Analyses

IL-2 secretion and RBL degranulation experiments were in triplicate and results were presented as mean ± SD. One way ANOVA with Bonferroni post-test was used for statistical comparisons; *P* values <0.05 were considered significant. All statistical analyses were conducted using GraphPad Prism v.4 software.

## Results

### The OX110 mAb Recognises CD200R in Some Mice Strains and Cross-reacts with CD200RLc

**Figure 2:**
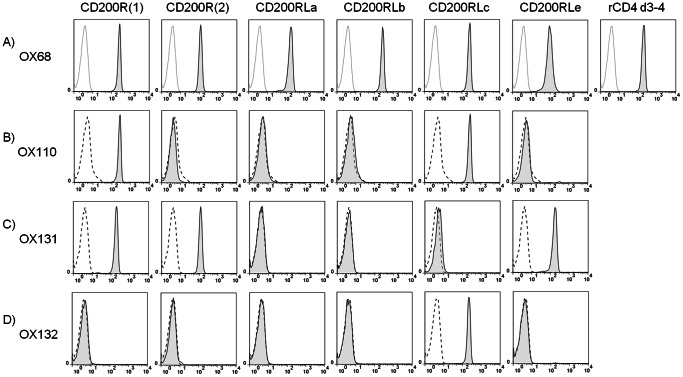
Specificity of OX110, OX131 and OX132 mAb. Streptavidin coupled magnetic beads were coated with biotinylated chimeric proteins containing extracellular domains of members of the mouse CD200R family and rCD4d3+4 and a biotinylation site (as indicated on top of each column). The binding of the mAb indicated in each row was analyzed by flow cytometry. (A) Protein coating levels for each group of magnetic beads were tested by staining with OX68 (CD4 mAb) (tinted solid line) or OX21 (control mAb) (thin line). Flow cytometry plot named rCD4 d3+4 indicates coating level for biotinylated rCD4d3+4 only. (B–D) OX110 (B), OX131 (C) and OX132 (D) mAb were used to stain magnetic beads coated with the chimeric proteins indicated above each column (tinted solid line), or control beads coated with biotinylated rCD4d3+4 only (dashed line). Data are representative of three experiments.

The heterogeneity in gene expression of the CD200R family between mice strains makes it important to know the specificity of reagents used to recognise them in case unexpected cross-reactions cause misleading results. We set up an assay consisting of magnetic beads coated with streptavidin and then chimeric proteins containing a site to enable the protein to be biotinylated, rat CD4d3+4 as a tag that can be recognised by OX68 mAb and the two extracellular domains corresponding to CD200R and all the activating receptors. Binding of mAb was followed by flow cytometry. The successful coating of the beads with the various recombinant proteins is shown (Fig. 2A) by labelling with OX68 mAb and FITC conjugated anti-mouse IgG. The OX110 mAb gives clear labelling of CD200R(1) as expected but failed to react with CD200R(2) - the second allele of the CD200R - and cross-reacted with the activating receptor CD200RLc (Fig. 2B). Ideally one would want mAb to see both CD200R alleles and not cross-react with activating receptors.

### New mAb Discriminate between CD200R and CD200RLc

Two new mAb, OX131 and OX132 were prepared by immunisation of rats with CD200R and CD200RLc respectively and screening on recombinant proteins and cell lines. These mAb were tested using the bead assay described above. Unlike OX110, OX131 mAb detected both CD200R isoforms and did not cross-react with the CD200RLc (Fig. 2C). However, this mAb cross-reacted with CD200RLe (Fig. 2C). CD200RLe is not present in most mice strains containing CD200R(1) [16]. Therefore, this antibody is still specific to CD200R when assayed in mice strains that lack CD200RLe such as C57BL/6. Finally, OX132 mAb recognised only CD200RLc, providing a specific reagent (Fig. 2D).

### Both Isoforms of CD200R Bind CD200

**Figure 3:**
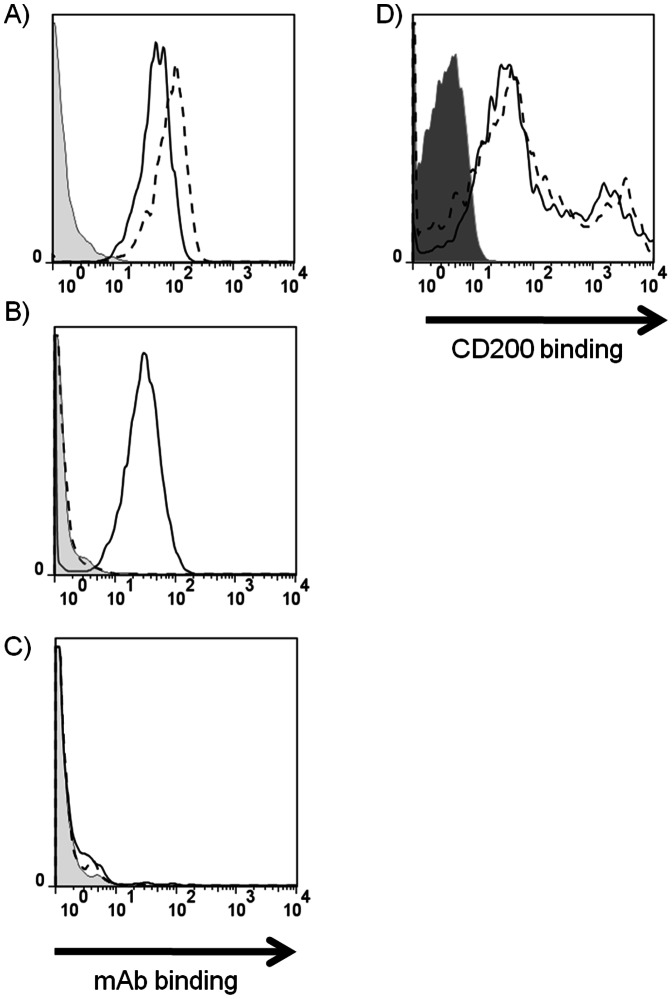
CD200R(1) and CD200R(2) differ in binding with CD200R mAb but not in binding with CD200. (A–C) Flow cytometry plots showing 2B4 Reay cell line stably transduced with CD200R(1) (A), CD200R(2) (B) or mock vector (C) which were stained with OX110 (dashed line), OX131 (solid line) or control antibody (tinted). (D) Flow cytometry plot showing the binding of CD200 coated fluorescent beads to 2B4 Reay cells transduced with CD200R(1) (solid line), CD200R(2) (dashed line) but not vector control (dark grey filled). The double peak in panel D is due to some clumping of the beads.

As the two alleles of CD200R differ in their sequence in the ligand binding domain and also show different mAb binding, the possibility that the proteins differed in ligand binding was tested. The 2B4 mouse T cell hybridoma cell line, which does not express CD200R (Fig. 3A), was transduced with retroviruses containing CD200R(1), CD200R(2) genes or empty vector (mock) (Fig. 3A, B, C). The observation above (Fig. 2) that OX110 mAb detected only CD200R(1) whilst OX131 recognised both CD200R(1) and CD200R(2) was confirmed. To test the ability of CD200R to bind CD200, recombinant protein consisting of the extracellular domains of mouse CD200 and rCD4d3+4 was immobilized onto streptavidin coated yellow fluorescent beads. Beads were then co-incubated with these cell lines and binding was assayed in flow cytometry (Fig. 3D). Both isoforms of CD200R bound the ligand CD200.

### OX131 but not OX110 mAb Blocks the CD200-CD200R Interaction

**Figure 4:**
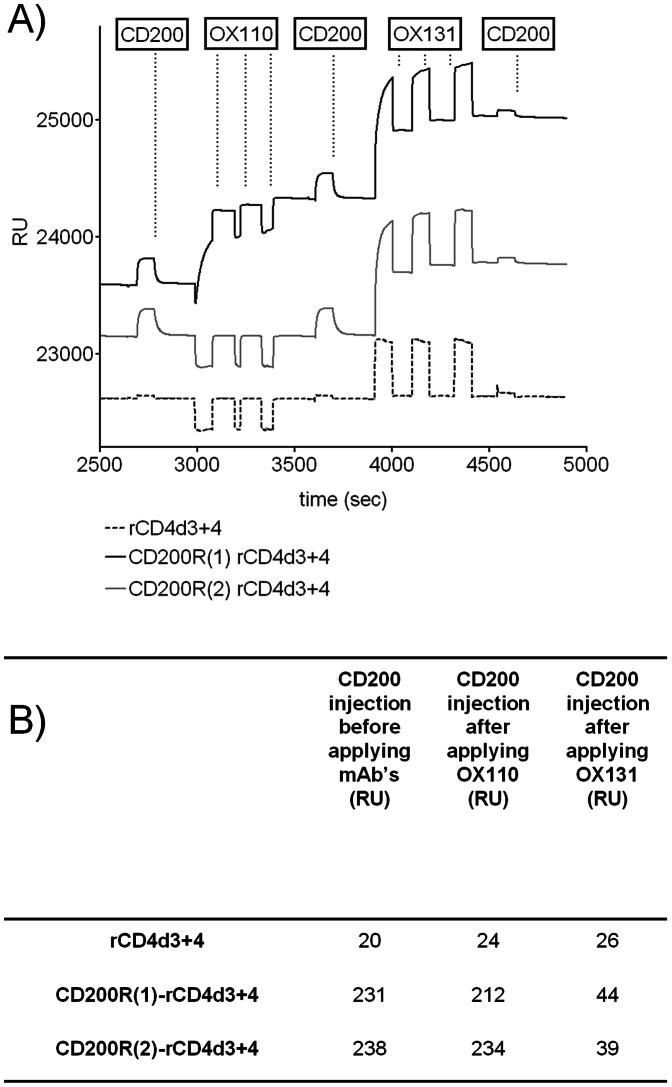
The CD200/CD200R interaction can be blocked by OX131 but not OX110 mAb. A) Biotinylated rCD4d3+4 (dashed line), CD200R(1) rCD4d3+d4 (solid black line) and CD200R(2) rCD4d3+4 (solid grey line) proteins were immobilized onto streptavidin coated CM5 chips (681, 726, 704 response units respectively). The changes in response units (RU) upon sequential injection of different soluble proteins (boxed and indicated by vertical dots) are shown. (Both antibodies were injected three consecutive times to ensure saturation on the immobilized proteins.) (B) Table showing the increase in response units upon injection of soluble CD200 compared to the pre-injection states for each flow cell. The values for the control rCD4d3+4 indicate the signal due to the high protein content of the CD200 sample.

It is important to know whether mAb inhibit ligand binding or not for functional analysis. OX110 and OX131 mAb are likely to see different regions of the CD200R as they show differential specificity for CD200R(1) and CD200R(2). The ability of OX110 and OX131 mAb to block the CD200R/CD200 interaction was tested by surface plasmon resonance. Biotinylated recombinant CD200R(1)-rCD4d3+4, CD200R(2)-rCD4d3+4 and rCD4d3+4 were immobilized onto a streptavidin coated chip at approximately equal levels. Soluble purified CD200 bound rapidly to both CD200R isoforms, reaching equilibrium at approximately equal levels and dissociated rapidly (Fig. 4A) as expected from the known affinity of the interaction [15]. This confirmed the cell binding experiments (Fig. 3D) that showed CD200 bound CD200R(1) and CD200R(2). The ability of CD200 to bind CD200R(1) and CD200R(2) after the addition of first OX110 and then OX131 mAb was tested. In each case the mAb was passed over in several injections to ensure saturation. As expected OX110 mAb bound only CD200R(1) and OX131 mAb bound both CD200R(1) and CD200R(2) (Fig. 4A). OX110 mAb had no effect on the ability of CD200 to bind to CD200R(1) whereas OX131 mAb blocked CD200 binding to both CD200R(1) and CD200R(2). The quantitative effects of the mAb on CD200 binding to the immobilized proteins are summarised (Fig. 4B).

### OX131 mAb Interaction with CD200R Prevents Inhibition by CD200

**Figure 5:**
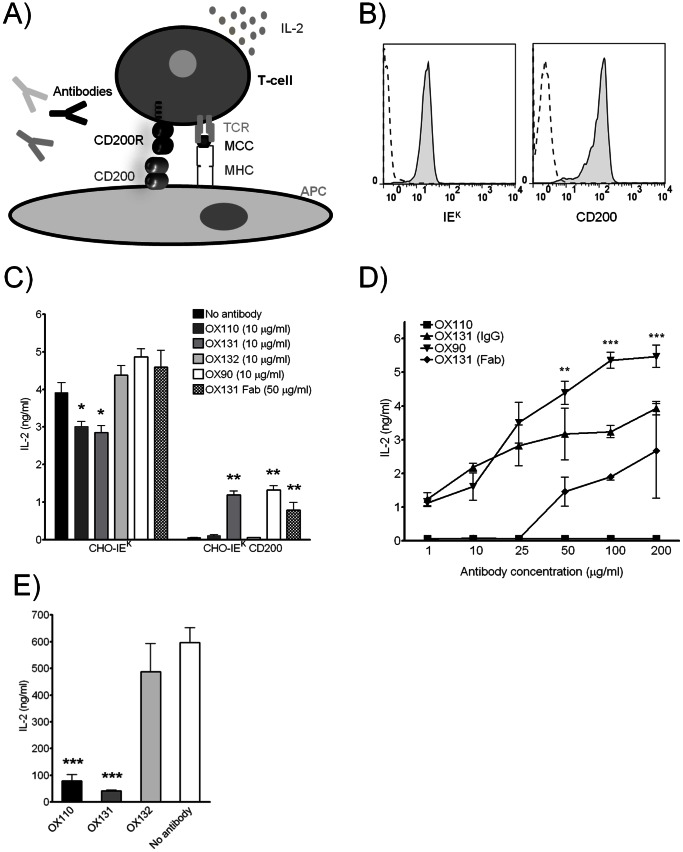
The effects of CD200R and CD200 mAb on T cell activation. (A) Schematic representation of the assay showing engagement of Moth Cytochrome C peptide (MCC), MHC-TCR and CD200/CD200R between the antigen presenting cell (APC) (CHO cells stably expressing IE^k^ and CD200) and 2B4 Reay T cells expressing CD200R(1) with IL-2 production as the readout. (B) **Left panel**: flow cytometry plots showing IE^k^ expression on CHO-IE^k^ CD200 cells (tinted solid line) compared to untransfected CHO cells (dashed line). **Right panel**: flow cytometry plots showing CD200 mAb staining of CHO IE^k^ CD200 cells (tinted solid line) compared to isotype control (dashed line). (C) Effect of different mAb on IL-2 release. Either CHO-IE^k^ cells **(left panel)** or CHO-IE^k^ CD200 cells **(right panel)** were used together with CD200R(1) expressing T cells and IL-2 concentrations were measured. Effects of mAb were statistically compared with no mAb applied condition (representative of more than seven experiments). (D) Changes in IL-2 secretion from CD200R(1) 2B4 Reay cells in response to increasing doses of mAb. CHO-IE^k^ CD200 cells as APC. IL-2 levels obtained with OX90 and OX131 mAb were compared (representative of three experiments). (E) 10 µg/ml of OX110, OX131, OX132 (control) or no mAb were immobilized onto plates together with 0.5 µg/ml of CD3 mAb. CD200R(1) expressing 2B4 Reay cells were incubated overnight and IL-2 release in each condition was assayed. IL-2 levels obtained by each mAb stimulated condition were statistically compared with no mAb stimulated control. (representative of three experiments). Statistically significant (p<0.05) results were shown with asterix. (*) indicates p<0.05, (**) indicates p<0.01 and (***) indicates p<0.001 (C–E).

The OX110 mAb has been shown to have agonistic inhibitory effects [35] presumably by cross linking the inhibitory receptor. As this mAb does not block the interaction (see above Fig. 4) one might expect different functional effects with the blocking OX131 mAb. This was tested using a T cell hybridoma expressing CD200R(1) (see Fig. 3B) and triggering by antigen presented on CHO cells expressing IE^k^. Addition of moth cytochrome C peptide provides a T cell activation assay leading to IL-2 production (Fig. 5A). The effects of CD200/CD200R engagement were tested by using a second antigen presenting cell line that had been transfected to express CD200 (Fig. 5A & B). The effects of the soluble mAb were first tested in the absence of CD200 on the normal CHO-IE^k^. High levels of IL-2 were produced and there was some reduction with OX110 and OX131 mAb but not with control mAb OX132, or OX90 (blocking mAb recognising CD200) (Fig. 5C left panel). Thus both of the CD200R mAb are giving inhibitory effects in the absence of the ligand. This suggests that CD200R triggering by receptor dimerization gave an agonistic inhibitory signal and this conclusion is supported by the lack of effect of Fab fragments of OX131. When CHO-IE^k^ expressing CD200 were used as antigen presenting cells, the IL-2 secretion was abolished (Fig. 5C right panel). This was due to the engagement of CD200R by CD200 on the antigen presenting cell as blocking this interaction with OX90 overcame some of this inhibition (Fig. 5C right panel). The blocking CD200R mAb, OX131, prevented the inhibition by CD200, whilst the non-blocking OX110 mAb had no effect (Fig. 5C).

These results showed that the TCR stimulus could be effectively neutralized by the CD200/CD200R interaction and OX110 mAb did not alter the interaction despite engaging with the receptor. On the other hand, using OX131 (blocking, CD200R mAb) or OX90 (blocking, CD200 mAb) generated significant IL-2 release by blocking the CD200/CD200R interaction (Fig. 5C right panel). However, even after blocking, the levels of IL-2 released were considerably lower than the conditions without CD200 on antigen presenting cells. This suggested that the mAb concentrations (10 µg/ml) were not sufficient to achieve complete blocking and the experiment was repeated using different concentrations of mAb. OX90 mAb gave dose dependent increases in IL-2 production to levels comparable to the normal CHO IE^k^ antigen presenting cells whilst the non blocking OX110 had no effect. OX131 mAb also gave strong relief of inhibition but not to the same degree as OX90. This is probably due to an opposing effect due to an agonistic inhibitory effect directly on the T cells (see below). Therefore OX131, while blocking the CD200/CD200R interaction and causing inhibition of the ligand induced stimulation of the CD200R, also dimerizes the CD200R and leads to an antibody induced stimulation of this receptor which generates slightly less efficient overall blocking of the CD200/CD200R interaction compared to OX90 (CD200 mAb) mediated blocking of the same interaction. Blocking the interaction with the monovalent OX131 Fab also gives inhibition albeit to a lesser extent that the divalent OX131 IgG (Fig. 5D).

The ability of the CD200R mAb to give agonistic inhibitory signals was tested in a T cell stimulation assay where CD200R(1) expressing 2B4 cells were stimulated with plate bound CD3ε and CD200R mAb at the same time. Both CD200R mAb (OX110 and OX131) gave almost complete inhibition of IL-2 secretion compared to that with control mAb (OX132) or no mAb (Fig. 5E). Much higher levels of CD200R stimulation by both antibodies were observed in Fig. 5E compared to Fig. 5C, D which is probably due to the difference between using immobilized plate-bound antibodies (Fig. 5E) and soluble antibodies (Fig. 5C, D). These results confirm the interpretation of the triggering abilities of both mAb in the antigen presentation dependent T cell stimulation setting (Fig. 5C, D). Thus OX110 and OX131 mAb have different functional effects, both can give agonistic signals (inhibition) but for OX131 mAb this can be offset by its ability to block the CD200/CD200R interaction.

### CD200RLc and CD200RLe mAb can give Activating Signals in Transfected Cell Lines

**Figure 6:**
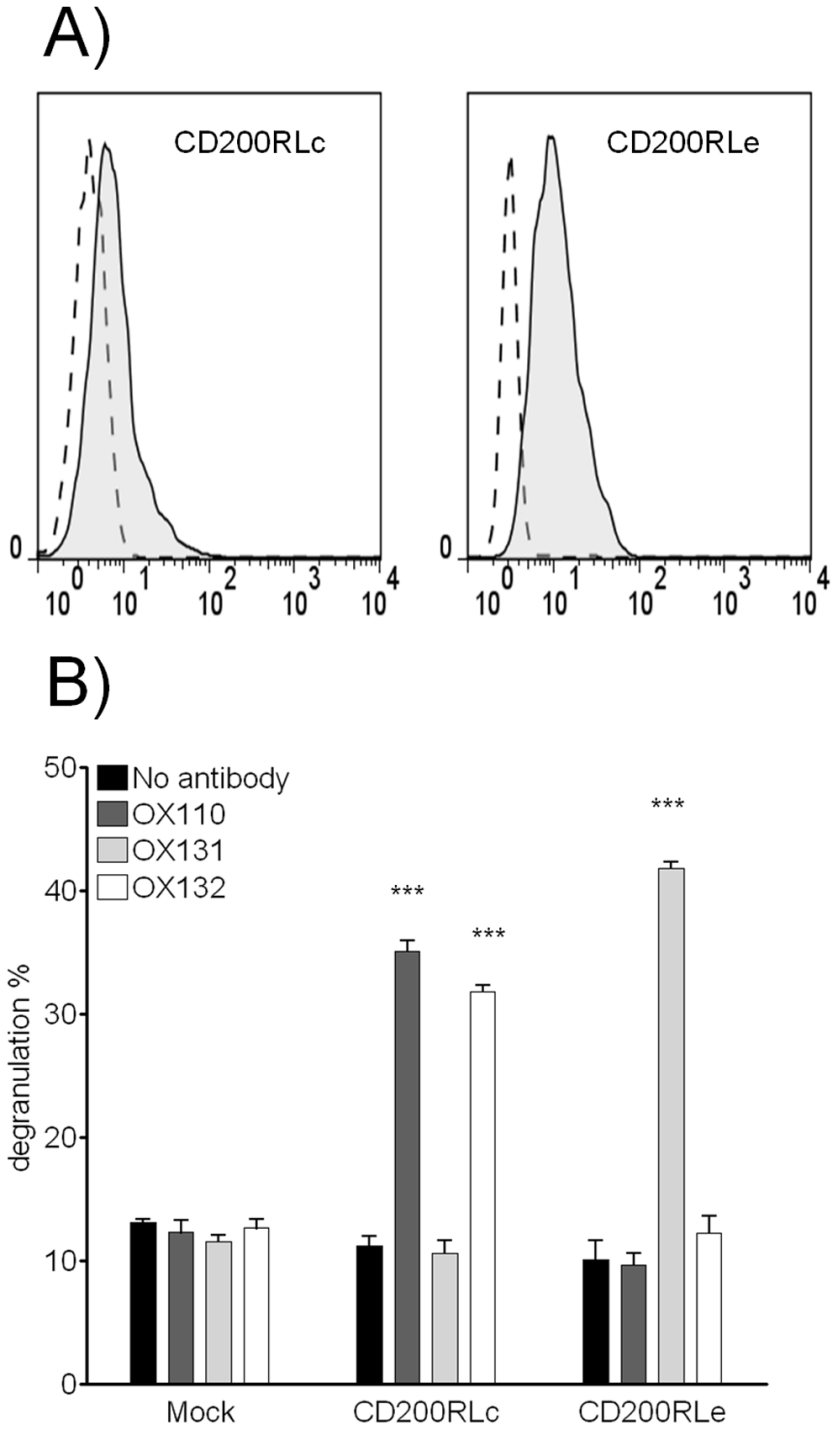
CD200RLc and CD200RLe can generate activating signals when triggered by specific mAb. (A) Flow cytometry plots showing expression of CD200RLc (left panel) and CD200RLe (right panel) (solid tinted lines) compared to the control mAb (dashed lines) on RBL.2H3 cells stably transduced with either CD200RLc or CD200RLe together with mouse DAP12. (B) RBL.2H3 cells, transduced with CD200RLc and DAP12, CD200RLe and DAP12 or mock vectors, were plated and soluble OX110, OX131, OX132 or no mAb were tested by overnight stimulation. The level of degranulation was measured by assaying β-hexosaminidase in the cell lysate and supernatants. Antibody stimulated groups were compared with no antibody control group with statistically significant results indicated by *** (p<0.001; representative of three experiments).

The activating members of the CD200R family, pair with DAP12 for surface expression [1], [2], [36] but activating potential has only been shown for CD200RLb [36]. We tested whether CD200RLc and CD200RLe triggering could result in cellular activation. The rat basophilic leukemia cell line RBL.2H3 was transduced with retroviral vectors harbouring CD200RL and DAP12 genes and surface expression of CD200RLc and CD200RLe confirmed by flow cytometry after staining with relevant antibodies (Fig. 6A). Cells were plated on flat bottom 96 well plates and stimulated overnight with media containing 10 µg/ml of mAb. Degranulation, assayed by hexosaminidase release, was used as the readout of cellular activation. Both CD200RLc and CD200RLe were shown to be capable of generating activating signals upon stimulation with relevant mAb (OX110 and OX132 for CD200RLc, OX131 for CD200RLe). This cellular activation setting also provided further proof that the antibodies can dimerize the receptors and give agonistic (inhibitory) as discussed previously (Fig. 5). Moreover, the absence of nonspecific stimulation by antibodies provided further functional evidence for specific binding abilities outlined in Figure 2.

### CD200R is Expressed on Myeloid Cells but not on Lymphocytes

**Figure 7:**
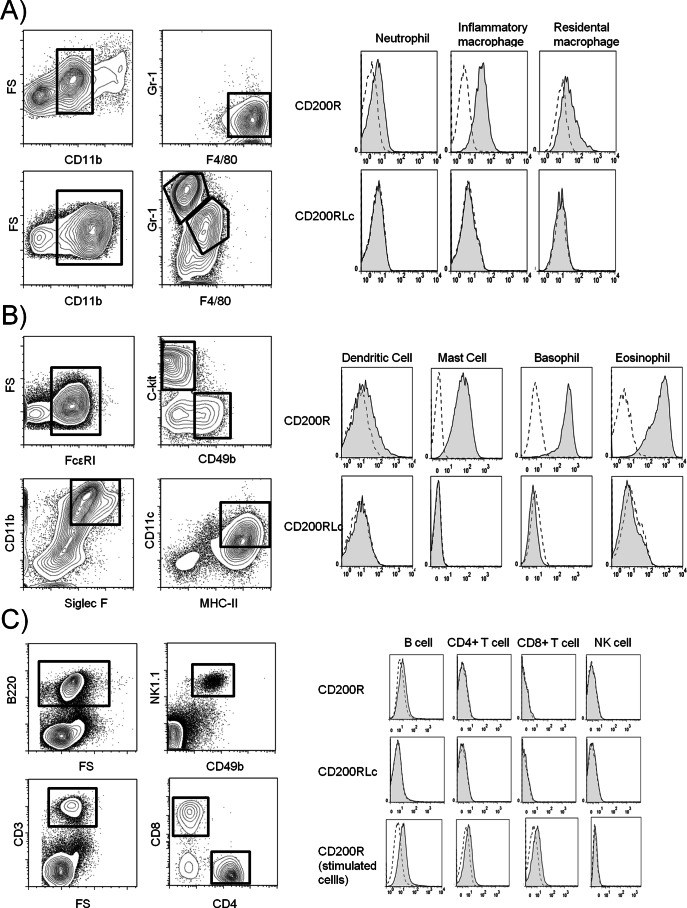
CD200R and CD200RLc expression on mouse leukocytes. (A) **Left panel:** Gating strategy for macrophage and neutrophils in peritoneal aspirates of naive (top) and zymozan stimulated (bottom) mice. For each mouse CD11b+ cells were gated in CD11b-forward scatter plot (left) then in these populations Gr-1(−)F4/80(+) population was gated as resident macrophages (top right), Gr-1(+)F4/80(−) population was gated as neutrophils (bottom right), Gr1(+)F4/80(+) population was gated as inflammatory macrophages (bottom right). **Right panel:** expression of CD200R (top row) and CD200RLc (bottom row) in macrophage and neutrophil populations (tinted solid lines) compared to control mAb (dashed lines). (B) **Left Panel:** Gating strategy for different *in vitro* cultures of mouse bone marrow cells. Cells grown for mast and basophil differentiation were first gated for FcεRI expression in FcεRI- forward scatter plot (top left). FcεRI(+) cells were further gated as C-kit(+)CD49b(−) mast cells (top right) and C-kit(−) CD49b(+) basophils (top right) in C-kit-CD49b plot. Cells cultured in IL-5 supplemented media were gated as CD11b(+)Siglec F(+) eosinophils in CD11b-Siglec F plot (bottom left). Cells cultured in GMCSF supplemented media were gated as CD11c(+)MHC II(+) dendritic cells in CD11c-MHC II plot (top right). **Right panel:** Expression of CD200R (top row) and CD200RLc (bottom row) in *in vitro* cultures of mouse bone marrow cells (tinted solid lines) (C) **Left panel:** Gating strategy for lymphocytes derived from mouse spleen. B cells were gated for B220 expression in B220-forward scatter plot (top left). NK cells were gated as double positives in NK1.1-CD49b plot (top right). T cells were first gated as CD3(+) population in CD3-forward scatter plot (bottom left), then this population was further gated into CD4(+) and CD8(+) cells in CD4/CD8 plot (bottom right). **Right panel:** Expression of CD200R in unstimulated splenocytes (top row), expression of CD200RLc in unstimulated splenocytes (middle row) and expression of CD200R in *in vitro* stimulated splenocytes (LPS for B cells, CD3 mAb for T cells and IL-2 for NK cells) (bottom row) are shown (tinted solid lines) compared to control mAb (dashed lines).

The availability of new reagents to discriminate between CD200R and CD200RLc (Table 1) allowed definitive tissue distributions of these proteins to be determined. Direct conjugates of F(ab)’_2_ fragments of the purified IgG were used to eliminate any possible Fc receptor binding. Although OX131 mAb cross-reacts with CD200RLe, it is specific for CD200R in C57BL/6 and BALB/c strains that lack CD200RLe (Table 1). All cells were also stained with the OX110 mAb which cross-reacts with CD200RLc so that any differences may indirectly indicate the presence of CD200RLc. Primary cells were obtained either directly from C57BL/6 mice or following inflammatory stimulation or by culture from bone marrow as described in Materials and Methods. CD200R was expressed on basophils, mast cells, eosinophils, macrophages and neutrophils (Fig. 7A & B). Dendritic cell culture showed partial expression (Fig. 7B) and no significant expression was shown on freshly isolated T cells, B cells and NK cells (Fig. 7C). *In vitro* stimulation of splenocytes with plate bound anti CD3 mAb or 1 µg/ml soluble LPS up-regulated the CD200R surface expression for T and B cells respectively. However, IL-2 mediated activation of splenocytes did not alter the absence of CD200R on NK cells (Fig. 7C). No difference was seen in staining by OX110 and OX131 mAb consistent with lack of CD200RLc that would be detected with OX110 but not OX131 mAb.

**Table 1:** Repertoire of CD200R family genes and predicted reactivity of mAb.

A. Repertoire of CD200R family genes						
Mouse strain	CD200R alleles		Activating receptor genes			
	(1)	(2)	RLa	RLb	RLc	RLe
Balb/c	+	−	+	+	+	−
C57BL/6	+	−	+	+	+	−
C3H/Hej	+	−	+	+	+	−
AKR	−	+	+	+	−	+
CD1	−	+	+	+	−	+
NOD	−	+	+	+	−	+
B. Reactivity of mAb						
mAb						
OX110	+	−	−	−	+	−
OX131	+	+	−	−	−	+
OX132	−	−	−	−	+	−

(A) The different genes present in common mice strains from genomic and biochemical analysis [1], [16]. (B) The predicted gene products recognised by the three mAb from this study.

### Surface Expression of CD200RLc Could not be Detected

The new OX132 mAb specific for CD200RLc was used to screen for the surface expression of this molecule. The same cell types screened for CD200R (see above) were stained with OX132 mAb F(ab)’_2_. CD200RLc expression could not be detected in any of the tested primary cells or the following cell lines including AC3, FSDC, RAW264.7 and P815 (formerly shown to have low levels of CD200RLc transcription in Q-PCR studies [2]). A variety of activation stimuli were tested on normal cells and cell lines but failed to give expression e.g. splenocytes stimulated with PMA-ionomycin, LPS, IFN gamma, CD3 mAb or IL-2, as well as RAW 264.7 and FSDC cell lines stimulated with IFN gamma, PMA, ionomycin, or LPS, P815 and AC3 cell lines stimulated with PMA, ionomycin or compound 48/80 (data not shown). The possibility that CD200RLc was expressed intracellularly was tested by staining after permeabilization of primary cells but no staining was obtained. To rule out the unlikely possibility that OX132 was not detecting CD200RLc on primary cells due to some modification, OX110 mAb was used to screen for the expression of CD200RLc in CD200R knock out mice where it should only detect this molecule but no labelling was obtained (data not shown).

## Discussion

Many of the therapeutic drugs being developed are either mAb or recombinant proteins reacting against the cell surface [37], [38]. Paired receptors are an increasingly important group of targets as indicated by CD200/CD200R in leukemia and CD47/SIRP in many cancers [18], [39], [40]. Paired receptors pose complications due to the variety of numbers of genes in individuals and the high degree of polymorphism [13] as illustrated with mAb against SIRPα where most mAb see only one of the common alleles [26]. With the identification of further alleles of CD200R like genes [2], [15], [16] and two alleles of CD200R that differ by seven amino acids in their extracellular region [16], a clear analysis of the specificity of CD200R mAb is essential in the interpretation of their functional effects. This is illustrated by the finding that OX110 mAb recognizes CD200R(1) in C57BL/6 mice with cross-reaction on CD200RLc although its expression is limited.

The distribution of CD200R was similar to that reported before in that it was expressed mainly on myeloid cells, mast cells and basophils [1], [4], [41] and also eosinophils that had not previously been tested. However we failed to find significant labeling of naive T and B cells as reported with a different mAb [1]. We did note T cell activation gave some induction of CD200R from very low levels in agreement with another study [42]. A trivial explanation seems unlikely as the same results were obtained with two mAb recognising different epitopes. CD200R was not expressed on rat T cells [6] although it was expressed on human T cells [1]. No cell surface expression of CD200RLc could be detected despite testing a wide range of cell types and stimuli and it is possible that it is only expressed under rare conditions. However OX132 mAb was shown to be specific for this receptor and also capable of triggering activating response upon engagement. Therefore, this mAb will be useful for future investigations on the biology of this poorly understood activating receptor.

Therapeutic mAb have the potential to act by various mechanisms such as agonistic signals, blocking of interactions, down regulation of receptors, elimination of cells by ADCC or other mechanisms [43]. In this study, we show that engagement of CD200R by mAb can give agonistic - inhibitory - signals as reported previously for CD200R mAb and also CD200-Fc fusion proteins [4], [44] and compatible with functional studies [35], [45]. However, blocking the interaction of CD200R with CD200 prevents the inhibition caused by ligand engagement. Therapeutic CD200 mAb are being evaluated for cancers where blocking the inhibitory signal may enhance the homeostatic mechanisms and give increased phagocytosis of the cancer or enhance immune responses against the cancer [10], [39]. In the functional studies described here both OX131 and OX110 mAb can give agonistic inhibitory signals when engaged but OX131 can give opposite effects due to blocking engagement by CD200. The Fab fragment of OX131 antibody, which retains the blocking capacity while being devoid of the receptor dimerization ability, could be an alternative blocking reagent to CD200 mAb upon being re-engineered for higher affinity to CD200R. However blocking the interaction with CD200 mAb is advantageous given the lack of variability in CD200 and that its short cytoplasmic region has no known signaling role.
